# A study on the accuracy of a new fluorescent detection method for vaginal fungi

**DOI:** 10.1186/s12905-022-02151-9

**Published:** 2022-12-30

**Authors:** Yuexia Chen, Wei Qu, Jianhong Tu, Fenfen Kong, Qinwen Jiang, Zhenghao Wang

**Affiliations:** Department of Pathology, The Third Hospital of Nanchang, Nanchang City, Jiangxi China

**Keywords:** Vaginal fungi, Liquid-based fungal method, Saline smear method, Fungal culture method

## Abstract

**Background:**

To investigate the positive rate and clinical applicability of liquid—based fungal method for detecting of vaginal fungi. We collect the secretions from the posterior vaginal fornix and the vaginal wall of 198 patients with clinically suspected fungi vaginitis patients for study.

**Methods:**

The vaginal fungi of vaginal discharge were detected by fluorescence method, i.e., by liquid—based thin-layer fungi fluorescence morphology staining detection kit (liquid—based fungal method), saline smear method and fungal culture method.

**Results:**

The positive rate of liquid-based fungal method, saline smear method was 50%, 25.75% respectively. The positive rate of liquid-based fungal method were 50%. The true positive rate of liquid-based fungal method (87.85%) was higher than that of saline smear method (45.79%, *P* < 0.001), which was easy to miss diagnosis. Moreover, the Kappa (K) of liquid-based fungal method was 0.81, and *P* < 0.01, which was statistically significant, indicating that the consistency of the two detection methods is good. Of the eight common symptoms of fungal vaginitis, the positive symptom coincidence rate of liquid-based fungal method was consistent with that of fungal culture method. It was also easier to see fungi under a microscope than with saline smear method.

**Conclusion:**

The liquid-based fungal method has a high positive coincidence rate and accuracy in the detection of vaginal fungi, and it is convenient to operate and implement steps. Therefore, it may be applied in clinical practice. Or a combination of several detection methods can be used.

## Introduction

Vaginitis is a common clinical gynecological disease caused by vaginal bacteria, vaginal fungi, *Trichomonas vaginalis* and other pathogens [1]. Among that, vulvovaginal candidosis (VVC), accounting for 20–45% of all vaginitis, is the second most common vaginal inflammatory disease only after bacterial vaginosis, and is mainly *Candida albicans* vaginosis, accounting for 85–90%, resulted in premenstrual vulva or vaginal itching and other systems [2]. The matrix metalloproteinase-8 (MMP-8) and fibroblast mediated proinflammatory immune response may be major factors causing symptoms [3, 4].

Especially in recent years, with the abuse of broad-spectrum antibiotics, the use of immune inhibitors, and the application of all kinds of gynecological treatment instruments, the incidence of fungal vaginitis is gradually rising [5–7]. Vulvovaginal candidiasis (VVC) can cause pain, extreme discomfort, mental distress, anxiety, altered self-esteem, impaired work performance, and interference with sexual and emotional relationships [8, 9]. Clinical diagnosis of fungal vaginal disease is very difficult, because the sings and symptoms of fungal vaginal disease is not peculiar to the disease, and the pathogeny resulting in similar symptoms may be diverse, such as bacterial vaginal disease,vaginal trichomoniasis, etc. In addition, *Candida albicans* detected in vagina do not meanVVC, because *candida* can also live in the vagina to coexist with the host and does not cause symptoms. Thus, the diagnosis of VVC requires a combination of clinical manifestations and laboratory confirmation of the presence of *candida*. The detection of mycelium, blastospores and spores in vaginal discharge is the detection standard for the diagnosis of fungal vaginosis[10]. As a result, it is a challenge to correctly diagnose fungal vaginosis because of limitations in the sensitivity and specificity of microbes in the laboratory detection. In a prospective study of the clinical diagnostic accuracy of bacterial vaginosis, trichomoniasis, and VVC in 535 women with vulvovaginal disease, Lowe et al. found that the diagnostic sensitivity and specificity of classical diagnostic methods (history, vaginal examination, pH, and microscopic examination of local preparations) were 83.8% and 84.8%, respectively [11].

Clinical common vaginal fungal detection methods include 10% potassium hydroxide (KOH) microscopic examination,Gram stain,wet mount microscopy method and fungal culture method (golden standard) [12–14]. The potassium hydroxide method was used to detect fungi in corneal scrapes, but the sensitivity varied widely, e.g. 94.3%, 81.0%, and 62.3% [15–17]. Gram stain method may result from the loss of lactobacilli by the process of fixation or Gram staining and takes a long time.Wet mount microscopy method is simple and fast, but their accuracy and sensitivity are unknown. DNA hybridization technology can detect vaginal fungi with sensitivity and specificity up to 96.3% [18]. If whole genome sequencing method is used, higher detection rate can be achieved [19]. However, these methods are cumbersome and expensive, which are not practical for routine clinical detection. The golden standard, fungal culture method also takes three to five days to produce results, which is too long.

Zhao et al. detected *Candida albicans* in the vagina of 110 patients with suspected VVC using saline KOH(potassium hydroxide) suspension method, CFW (Calcofluor White), FB 85 (fluorescent brightener 85) method and fungal culture method respectively, and concluded that CFW had the highest sensitivity, specificity and accuracy [20]. Previously, CFW has been commonly used by many researchers to detect the presence of skin fungi, and this was the first time to use CFW to detect vaginal fungi [21].

Based on CFW [22], this study carried out vaginal discharge fungi detection for 198 gynecological outpatients, by liquid—based thin-layer fungi fluorescence morphology staining detection kit (liquid-based fungal method). which relies on the combination of fungal fluorescent staining solution in the kit and beta cell wall polysaccharides (such as chitin and cellulose, etc.) in the samples to mark the fluorescent material. Under the specific excitation light band (340–400 nm) of fluorescence microscope, fungal myceliume or spores can emit blue-green fluorescence, which is easier to identify than the traditional method, having simple operation and fewer steps. Again, it is faster to obtain results, having higher sensitivity and accuracy. In addition, the enrichment effect of the kit was further achieved by liquid—based thin-layer preparation method on the basis of CFW method to reduce the rate of missed detection. However, there are also shortcomings. For instance, the liquid—based thin-layer preparation fungi fluorescence staining detection kit(liquid-based fungal method) is only a qualitative detection of fungal infection, which can only identify fungal and non-fungal, but cannot identify the type of fungi.

## Materials and methods

The vaginal discharge of 198 patients with suspected fungal vaginosis from October 2020 to February 2021 in Nanchang Third Hospital were collected and detected by different methods respectively.

### Patient screening

According to the study, patients who experience either (1) vulva itching, (2) peculiar smell, (3) increasing vaginal discharge, (4) frequent urination, (5) painful urination, (6) local erosion, (7) increasing vaginal discharge with bean dreas-like secretions,(8) lent erythema, edema, and scratches shaped like geographic pattern suffer from one or some of the symptoms.

### Experimental methods

The vaginal discharge detected for fungi of each screened patient were carried out by three methods, saline smear method, liquid-based fungal method.The vaginal discharge were collected by three sterile cotton swabs: The vaginal discharge from the first cotton swab were evenly coated on the slide, and then saline was dropped for microscopic examination (Olympipas, biological microscope, CX23LEDRFSIC). If hypha were found under the microscope, it was considered as fungi positive. The pictures were taken by Huawei mobile phone P10. The second cotton swab was immersed in a vial of liquid based cell and microbial treatment preservation reagent type II (Jiangxi Yeli, YL-FPS-I), then making slides by the equipment(Nanjing Jianang, JY-8000 A), 50 µl fluorescent staining solution (Yl-FPS-I) was dropped onto the slide and observed under a fluorescence microscope. If the hyphae or spores or blastospore showed strong fluorescence, it was considered as fungi positive. The third sterile cotton swab was inoculated on an AGAR plate. The AGAR plate was incubated at 35 ℃ for 72 h, and the colonies were observed after 72 h. If colonies were observed, it was considered as fungi positive.

### Statistical analysis

SPSS 22.0(IBM SPSS Statistics) software was used to analyze all data, and Chi-square test,Kappa (K) test and McNemar (M) test method were adopted. When Kappa ≥ 0.75, the consistency of the two is good; when 0.75 > Kappa ≥ 0.4, the consistency of the two is general, whereas when Kappa < 0.4, the consistency of the two is poor. In addition,* P* < 0.05 indicates that the difference is statistically significant.

## Results

### Results of vaginal fungi detection by various methods

Results of liquid-based fungal method are shown in Table 1. Among the 198 patients collected, 99 patients were positive and 99 patients were negative for vaginal fungi detected by liquid-based fungal method. That is, the positive rate and negative rate were 50% respectively (Table 1). While the saline smear method only detected 51 positive patients, with the positive rate of only 25.75%, and the rest 147 patients was negative, with the negative rate as high as 74.26% (Table 2).

**Table 1:** Vaginal fungal results detected by liquid-based fungal method

Method		Fungal culture method (golden standard)		Total (n)
		+	−	
Liquid-based fungal method	+	94	5	99
	−	13	86	99
Total (n)		107	91	

**Table 2:** Vaginal fungal results detected by Saline smear method

Method		Fungal culture method (golden standard)		Total (n)
		+	−	
Saline smear method	+	49	2	51
	−	58	89	147
Total (n)		107	91	

### Comparison of detection efficiency between different detection methods and golden standard culture method

As shown in Table 3, the true positive rate of liquid-based fungal method (87.85%) was higher than that of saline smear method (45.79%, *P* < 0.001), which was easy to miss diagnosis. The results in Table 4 showed that there was no difference in the true negative rate between the liquid-based fungal method (94.5%) and saline smear method (97.8%,* P* = 0.248). The results showed that the liquid-based fungal method was sensitive, but the positive rate of saline smear method was low.

**Table 3:** True positive rate comparison between liquid-based fungal method and Saline smear method

Methods	+	−	Total (n)	True positive rate	*χ*^2^	*P*
Liquid-based fungal method	94	13	107	87.85%	42.68	< 0.001
Saline smear method	49	58	107	45.79%

**Table 4:** True negative rate comparison between Liquid-based fungal method and Saline smear method

Methods	+	−	Total (n)	True positive rate	*χ*^2^	*P*
Liquid-based fungal method	5	86	91	94.5%	1.337	0.248
Saline smear method	2	89	91	97.8%		

Taking fungal culture method as the golden standard for comparison, the positive coincidence rate, negative coincidence rate and accuracy of liquid-base fungal method were 87.85%, 94.51% and 90.91%, respectively, and Kappa (K) was 0.81,* P* < 0.01, which was statistically significant, indicating good consistency between the two detection methods. While McNemar (M) was 0.09, indicating that the McNemar (M) test methodhas no statistical significance and there is no difference between the two detection methods. Again, the results of the two statistical methods are opposite. Such contradictory results indicate that the two statistical methods have inconsistent use of information, and Kappa test would use the information used in contingency tables. However, McNemar test only uses the information on non-diagonal cells, that is, it only concerns about the inconsistent evaluation information between the two methods. Therefore, when there is a contradiction between the two, Kappa value is mainly referred to, so that the liquid-based fungal method is in good consistency with the golden standard fungal culture method (Table 5).

**Table 5:** Comparison of detection index between different detection methods and fungal culture method (golden standard)

Method	Positive coincidence rate	Negative coincidence rate	Accuracy	Kappa (K)	McNemar (M)	*P* value
Liquid-based fungal method	87.85	94.51	90.91	0.81	0.09	< 0.01
Saline smear method	45.79	97.80	69.70	0.417	0.01	< 0.01

The positive coincidence rate, negative coincidence rate and accuracy of saline smear method were 45.79%, 97.80% and 69.70% respectively, and Kappa (K) was 0.417, *P* < 0.01, which was statistically significant, indicating that the consistency of the two detection methods is general. While McNemar (M) < 0.01, indicating that the McNemar (M) test method is statistically significant, and the two detection methods are different. These results indicate that the consistency between saline smear method and golden standard fungal culture method is poor. In addition, the return rate of patients with liquid—based fungal method was 91.5% higher than that of patients with saline smear method (Table 5).

### Analysis of coincidence rate between different detection methods and clinical symptoms.

According to the statistical results of this study, eight common symptoms upon (1) vulva itching, (2) peculiar smell, (3) increasing vaginal discharge, (4) frequent urination, (5) painful urination, (6) local erosion, (7) increasing vaginal discharge with bean dreas-like secretions, (8) lent erythema, edema, and scratches shaped like geographic pattern Patients with fungal vaginitis had the highest association with symptom 7, followed by symptom 1 and symptom 5. In addition, the positive symptom coincidence rate of liquid-based fungal method was consistent with that of fungal culture method, perhaps this data can assist clinicians in diagnosis and prediction (Table 6).

**Table 6:** Correlation analysis between different detection methods and different clinical symptoms

Symptoms	Total	Cases (n)			Positive coincidence rate of symptoms		
		Saline smear method	Liquid-based fungal method	Fungal culture method	Saline smear method	Liquid-based fungal method	Fungal culture method
Symptom 1	94	27	56	49	28.72%	59.57%	52.13%
Symptom 2	24	1	12	6	4.17%	50.00%	25.00%
Symptom 3	99	21	50	36	21.21%	50.51%	36.36%
Symptom 4	0	0	0	0	0	0	0
Symptom 5	2	0	0	1	0	50.00%	50.00%
Symptom 6	0	0	1	0	0	0	0
Symptom 7	27	19	25	25	70.37%	92.59%	92.59%
Symptom 8	56	22	43	54	39.28%	78.57%	96.43%

**Fig. 1:**
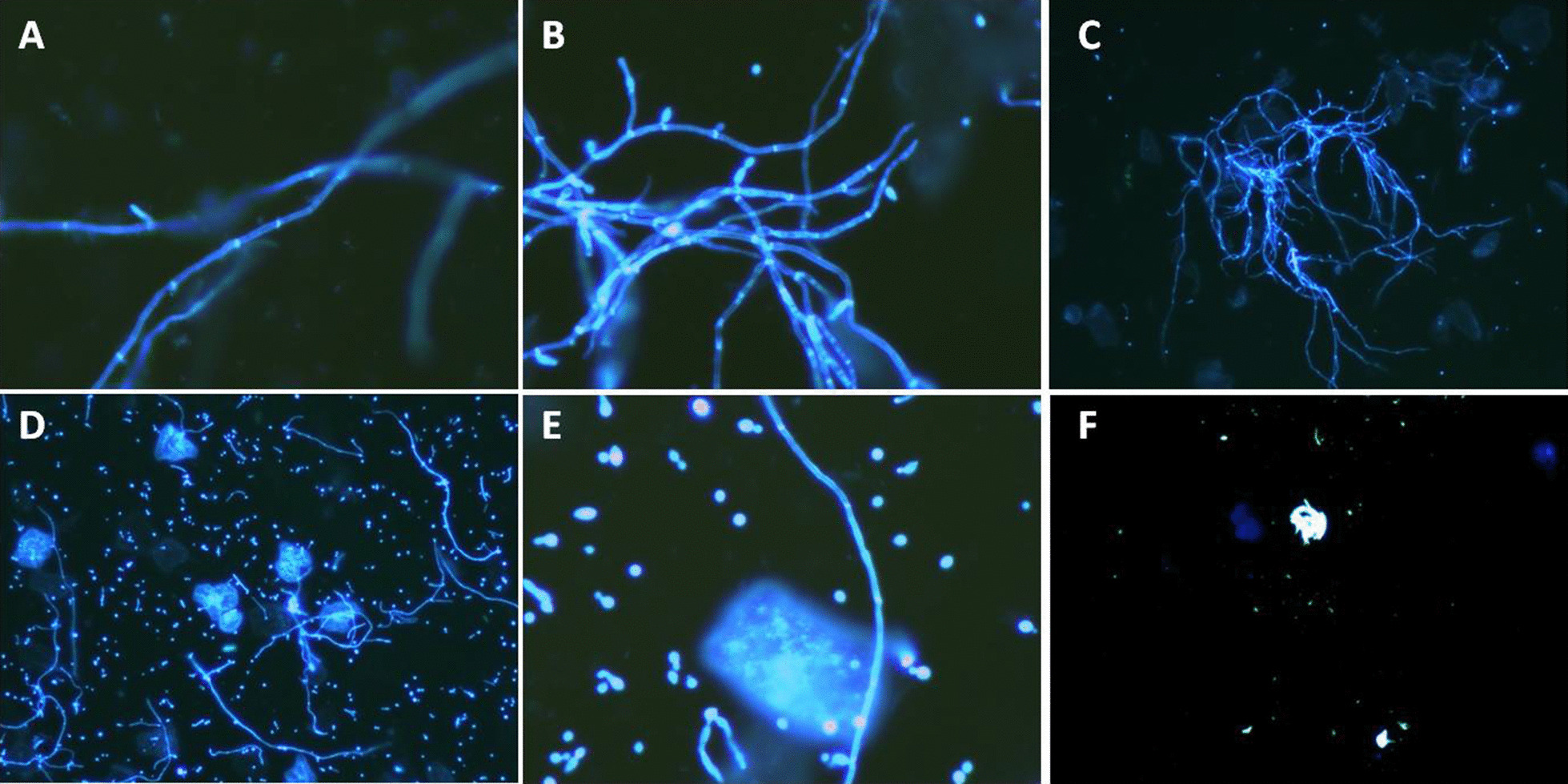
The images under fluorescence microscopy with liquid—based thin-layer preparation fungi fluorescencestaining method. **A** Hypha, **B**, **C** hyphae and spores, **D**–**E** hyphae, spores and blastospore, **F** fungi nagetive

**Fig. 2:**
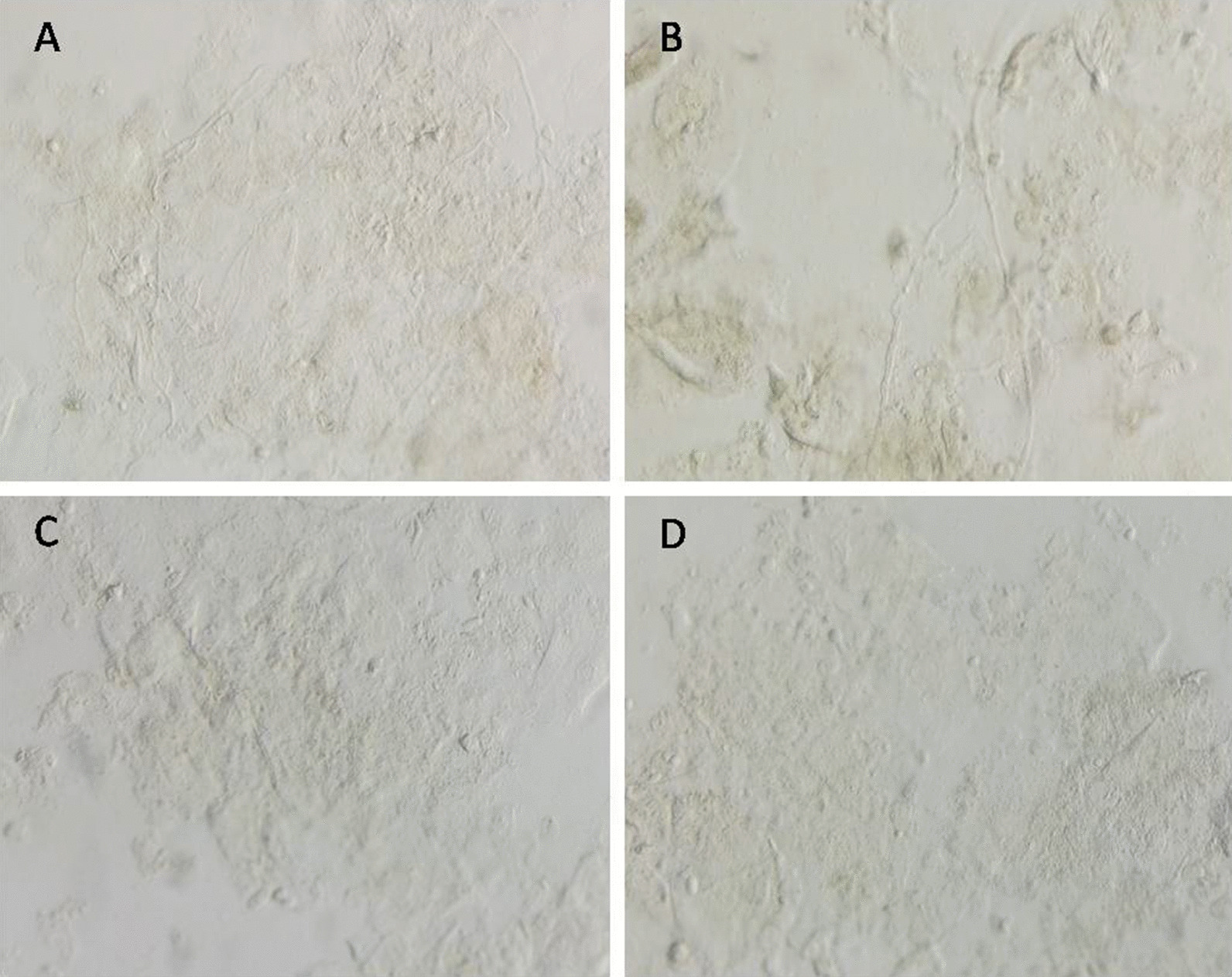
The images under polarized light microscopy of Saline smear method. **A**, **B** Hypha, **C**, **D** fungi nagetive

Liquid-based fungal method can be intuitively shown by fluorescence microscope, and fungal spores, mycelium and blastospore can be easily identified in the positive vaginal fungi patients, while the negative vaginal fungi patients can only be seen as dark under the microscope (Fig. 1). On the other hand, it is difficult to observe transparent mycelium and spores in the saline smear method through polarizing microscope, and there are many other components in the background, such as white blood cells and vaginal exfoliated epithelium, etc. having interfered the detection, which may easily lead to misdiagnosis and false positive or false negative results (Fig. 2). Therefore, in terms of the ease degree of looking for fungi under the microscope, the liquid-based fungal method is obviously superior to the saline smear method.

## Discussion

The microflora structure of female vaginal microecosystem is complex, and *lactobacillus* is the dominant flora in the vaginal microflora under physiological conditions [23, 24]. *Candida* is a polymorphic fungi whose morphologic transformation is an important mechanism of human host disease. Immune deficiency, destruction of epithelial integrity and microecological imbalance are common pathogenic factors [25]. Asymptomatic vaginal colonization patients with normal immunity do not require any treatment, whereas symptomatic patients require treatment, such as azole, polyene and ciclopirox olaminecan be selected [26–29]. Most *candida* are sensitive to universal antifungal agents, and 75–90% of cases can be successfully treated with topical antifungal therapy, mainly with clotrimazole or nystatin cream. However, if the infection persists, systemic treatment (e.g., fluconazole) may be used.

In this study, vaginal discharge were collected from 198 gynecological patients and targeted samples were taken from the same patient.The positive coincidence rate, negative coincidence rate and accuracy among liquid-based fungal method, saline smear method, golden standard fungal culture method were compared, so as to verify the feasibility in the clinic determination of liquid-based fungal method.

In this study, 99 cases of positive and negative vaginal fungal patients were detected by liquid-based fungal method, that is, the positive and negative rates were 50% respectively. The positive coincidence rate, negative coincidence rate and accuracy of liquid-base fungal method were 87.85%, 94.51% and 90.91%, respectively. And Kappa (K) was 0.81, *P* < 0.01, which was statistically significant, indicating that the consistency of the two detection methods was good. We found that the positive coincidence rate of symptoms of the liquid-based fungal method was higher than that of the fungal culture method. In addition, under the fluorescencemicroscope, it was found that the liquid-based fungal method could detect the existence of fungal spores, mycelium and blastospores intuitively and quickly by contrast, which was easier to diagnose.

Yunzhuan Zhao et al. detected *Candida albicans* in the vagina of 110 patients with suspected VVC using saline KOH suspension method, CFW, FB 85 method and fungal culture method respectively, and concluded that CFW had the highest sensitivity, specificity and accuracy [20]. In their study, the sensitivity, specificity and accuracy of CFW were 92.2%, 100% and 84.5%, respectively, and the sensitivity, specificity and accuracy of similar FB 85 method were 88.3%, 100% and 91.8%, respectively. In this study, the positive coincidence rate, negative coincidence rate and accuracy of liquid-based fungal method were 87.85%, 94.51% and 90.91%, respectively, which were similar to the results of Yunzhuan Zhao et al., further verifying the feasibility of fluorescence method for detection of vaginal fungi. However, the liquid-based fungal method has some shortcomings. It is only a qualitative detection of fungal infection, which can only identify fungal and non-fungal, but can not identify the type of fungi. Therefore, if conditions permit, the combined detection of vaginal fungi by liquid-based fungal method, saline smear method and fungal culture method may also be a good choice.

## Conclusion

To sum up, the liquid-based fungal method have higher positive coincidence rate and accuracy in terms of vaginal fungal detection, and it is also easy observe under microscope. Therefore, it may be carried out in clinical application relying on simple and quick operation to not only shorten the testing time, but also guarantee the accuracy of test results. Moreover, its price is economical, which can be accepted by many patients. If several detection methods are used together, it may be a better choice.

## Data Availability

All data are included in the manuscript.

## Abbreviations

Liquid-based fungal method: Liquid—based thin-layer fungi fluorescence morphology staining detection kit
VVC: Vulvovaginal candidosis
